# Heart-Shaped Infarct on MRI and Its Implications in Bilateral Medullary Syndrome

**DOI:** 10.7759/cureus.70361

**Published:** 2024-09-27

**Authors:** Prem Balaji Reddy Lankapothu, Sathish Kumar, Sharath Chandra Dasi, Shrinidhi Bhaskaran, Arun Kumar Bathena

**Affiliations:** 1 General Medicine, Saveetha Medical College and Hospitals, Saveetha Institute of Medical and Technical Sciences, Saveetha University, Chennai, IND; 2 Neurology, Saveetha Medical College and Hospitals, Saveetha Institute of Medical and Technical Sciences, Saveetha University, Chennai, IND; 3 Internal Medicine, Saveetha Medical College and Hospitals, Saveetha Institute of Medical and Technical Sciences, Saveetha University, Chennai, IND

**Keywords:** acute quadriplegia, bilateral, heart shape, medial medullary syndrome, medullary infarction, mri images, vertebral artery

## Abstract

Bilateral medullary syndrome (BMS) is an extremely rare and devastating neurological disorder resulting from ischemia or infarction of the medulla oblongata. This case report presents two unique instances of BMS, both leading to fatal outcomes.

Case 1 describes a 70-year-old male with a history of systemic hypertension who presented with limb weakness, slurred speech, and dysphagia. An MRI revealed a heart-shaped infarct in the medulla, confirming the diagnosis of BMS. Despite medical intervention, the patient developed quadriplegia and respiratory failure due to aspiration pneumonia and ultimately succumbed to his condition.

Case 2 involves a 40-year-old male with poorly controlled diabetes and hypertension, presenting in an unresponsive state after a suspected seizure. MRI findings confirmed bilateral medial medullary stroke. The patient’s condition deteriorated in the intensive care unit (ICU), complicated by a urinary tract infection and systemic inflammatory response syndrome (SIRS), leading to his demise.

These cases underscore the complexity and severity of BMS, highlighting the need for prompt diagnosis and multidisciplinary management. The heart-shaped infarct seen in Case 1 serves as a radiological hallmark, while Case 2 emphasizes the importance of identifying underlying risk factors such as protein C deficiency. Despite advanced imaging techniques and supportive care, the prognosis for BMS remains poor, with high mortality rates. Further research into novel therapeutic approaches is warranted to improve outcomes in this rare and challenging condition.

## Introduction

Medullary syndrome, also known as medullary infarction, is a rare neurological condition with an incidence rate of 0.5-1.5% in all ischemic strokes, characterized by the infarction of structures within the medulla oblongata of the brainstem [1,2]. Bilateral medullary syndrome (BMS) is an exceptionally rare neurological condition characterized by ischemic or infarct lesions involving both sides of the medulla oblongata, a critical part of the brainstem responsible for autonomic functions such as breathing, cardiovascular regulation, and motor control [3,4]. The medulla's central role in maintaining these vital functions makes infarctions in this area particularly life-threatening. BMS typically results from bilateral occlusion of vertebral arteries or their branches, leading to ischemia in the medullary pyramids and surrounding structures [5-8].

Clinically, BMS presents with a wide range of symptoms, including quadriplegia, sensory deficits, cranial nerve dysfunction, and dysautonomia, complicating both diagnosis and management [9,10]. While magnetic resonance imaging (MRI), particularly with diffusion-weighted imaging (DWI), plays a crucial role in diagnosis; the rarity and varied clinical presentation of BMS often lead to delayed recognition. The unique finding of a heart-shaped infarct on MRI has emerged as a radiological hallmark of this condition, though its occurrence is exceedingly uncommon [7-13].

The heart-shaped infarct observed in this patient’s MRI is a unique finding that likely represents the distribution of blood flow and the anatomical configuration of the medullary arteries, serving as a radiological clue in diagnosing BMS. This case report presents two unique and fatal instances of BMS, illustrating the challenges of early diagnosis and management. Both cases emphasize the importance of advanced imaging for accurate diagnosis and highlight the grave prognosis associated with this condition, despite aggressive therapeutic interventions. Given the high mortality and limited therapeutic options, further investigation into the underlying pathophysiology and potential treatment strategies for BMS is critical to improving patient outcomes.

## Case presentation

Case 1

A 70-year-old male from a middle-class family, with a known history of systemic hypertension and intermittent alcohol use, presented with complaints of weakness in the right upper and lower limbs following an episode of dizziness. He also experienced slurred speech and difficulty swallowing. Upon arrival, the patient was conscious but disoriented.

On examination, the patient had a National Institutes of Health Stroke Scale (NIHSS) score of 18 on admission, a blood pressure of 160/90 mmHg, a pulse rate of 95 bpm, and a random blood sugar level of 124 mg/dL. Motor examination revealed bilateral weakness in the lower extremities with 0/5 power on both sides and upper limb weakness initially at 3/5, which progressed to 0/5 within five hours. Deep tendon reflexes were diminished. Cranial nerve examination showed bilateral hypoglossal nerve palsy, resulting in no tongue movement, palatal weakness, and an impaired gag reflex. Sensory examination revealed a loss of sensation to pain and temperature. There were no signs of ptosis or miosis and anhidrosis. Extraocular movements with horizontal movement restrictions are noted. A Ryle's tube was inserted as a precaution against aspiration. A non-contrast CT scan of the head showed no acute hemorrhage or mass effect. Due to a high suspicion of brainstem pathology, an MRI of the brain with DWI and apparent diffusion coefficient (ADC) mapping was performed.

**Figure 1:**
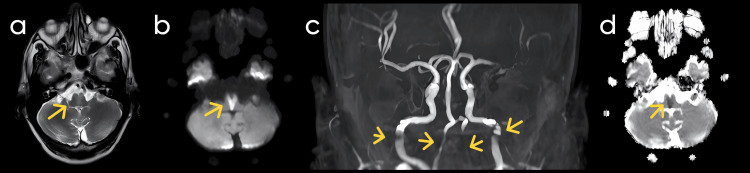
MRI Sequences of Case 1 (a) FLAIR (fluid-attenuated inversion recovery) imaging revealed hyperintensity involving the bilateral pyramids and central medulla. (b) DWI (diffusion-weighted imaging) demonstrated areas of diffusion restriction. (c) MR (magnetic resonance) angiogram showed flow restriction in both vertebral arteries and the basilar artery, with no evidence of dissection or occlusion. (d) ADC (apparent diffusion coefficient) mapping confirmed diffusion restriction in the affected region.

The MRI revealed T2/fluid-attenuated inversion recovery (FLAIR) hyperintensity affecting the bilateral pyramids and central medulla, confirming a heart-shaped infarct in the medulla and the diagnosis of BMS (Figure 1a) and a distinct area of diffusion restriction DWI (Figure 1b) with corresponding low ADC (Figure 1d). The involvement of the basilar artery and horizontal gaze restriction are suggestive of locked-in syndrome. The unique infarct shape suggested vascular involvement, likely linked to atherosclerotic changes in the vertebral arteries. An MR angiogram indicated flow restriction in both vertebral arteries and the basilar artery, without evidence of dissection or occlusion (Figure 1c).

During hospitalization, the patient's weakness progressed to all four limbs, resulting in quadriplegia, and he developed aphasia. Due to ongoing micro-aspiration, the patient experienced respiratory distress secondary to aspiration pneumonia, requiring intubation. Initial routine blood tests revealed anemia with a hemoglobin level of 8.2 g/dL, deranged renal function with urea at 82 mg/dL, creatinine at 3.2 mg/dL, and uric acid at 7.7 mg/dL. The coagulation profile and glycated hemoglobin (HbA1c) were within normal ranges. There was no dyslipidemia. Further work-up, including echocardiography, ruled out cardiac embolism, while an ultrasound showed bilateral shrunken kidneys with loss of corticomedullary junction, leading to a diagnosis of underlying chronic kidney disease (CKD). Despite elevated inflammatory markers, there was no initial evidence of infection or autoimmune disease.

Later in the course, the patient’s white blood cell count increased to 25,500 cells/µL, indicating leukocytosis, and renal function deteriorated further. A chest X-ray revealed bilateral opacities suggestive of aspiration pneumonia, and cultures of endotracheal secretions grew *Acinetobacter baumannii.* Treatment included dual antiplatelets (Ecosprin 75 mg and clopidogrel 75 mg), statins (atorvastatin 40 mg), antibiotics, and supportive care. Despite all medical interventions, including management of elevated white blood cell counts, hypotension, and reduced respiratory effort, the patient's condition continued to deteriorate, ultimately resulting in his death.

Case 2

A 40-year-old male was brought to the emergency department in an unresponsive and drowsy state, suspected of having a new-onset seizure. He was found unconscious on the bathroom floor after the door was forced open. While there were no witnesses to any convulsive activity, a history of loss of consciousness was reported.

The patient had a medical history of poorly controlled diabetes mellitus and systemic hypertension, both diagnosed three years prior, with poor adherence to medication over the past year. He also had a history of a cerebrovascular accident (CVA) involving the pons, for which he was on antiplatelet therapy and statins (Ecosprin 75 mg and atorvastatin 10 mg) and was able to perform his daily activities prior to this event. On initial assessment, the patient’s Glasgow Coma Scale (GCS) was E4 (eyes open spontaneously), V2 (incomprehensible sounds), and M1 (no motor response), and an impaired gag reflex necessitated immediate intubation for airway protection. His vital signs showed a blood pressure of 180/100 mmHg, pulse of 93 bpm, and oxygen saturation of 98% on mechanical ventilation in pressure control mode. The NIHSS score for this patient is 11. Neurological examination revealed bilateral lower limb weakness (power 0/5) and upper limb weakness (power 0/5). Deep tendon reflexes were diminished, and bilateral plantar reflexes were extensor. Cranial nerve examination is unable to elicit; however, the pupils were reactive to light, with no signs of miosis observed. Extraocular movements could be assessed. Arterial blood gas (ABG) revealed elevated lactate levels (6.2 mmol/L), raising suspicion of a seizure episode, and anti-epileptic (levetiracetam 500 mg BD) treatment was initiated. Further investigations showed a normal hemogram, renal, and liver function tests, and a normal urine routine. There was no evidence of dyslipidemia, but his HbA1c was significantly elevated at 13.5 when treated with insulin. Electrolyte levels, including calcium and magnesium, were normal.

**Figure 2:**
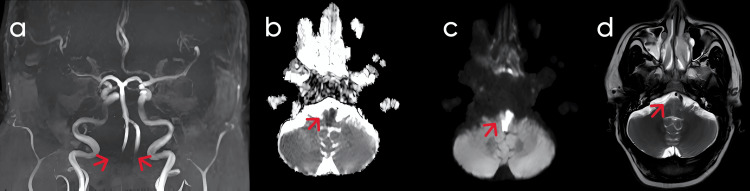
MRI Sequences of Case 2 (a) MR (magnetic resonance) angiography showed flow restriction in both vertebral arteries. (b) ADC (apparent diffusion coefficient) mapping confirmed diffusion restriction, consistent with acute infarction. (c) DWI (diffusion-weighted imaging) revealed areas of restricted diffusion in the affected regions. (d) FLAIR (fluid-attenuated inversion recovery) imaging showed hyperintensities in the medial aspect of the bilateral medulla oblongata.

A CT scan of the brain showed small vessel ischemic changes in the right frontal lobe and a chronic lacunar infarct in the left gangliocapsular region. MR angiography showed flow restriction in both vertebral arteries (Figure 2a). MRI of the brain revealed focal areas of diffusion restriction DWI (Figure 2b) with low ADC (Figure 2c) in the medial aspect of the bilateral hemi medulla, with corresponding T2/FLAIR hyperintensities (Figure 2d), T1 hypo intensities, and chronic infarct with gliosis and encephalomalacia in the right frontal lobe. An EEG was performed and showed no abnormalities. The patient was diagnosed with bilateral medial medullary stroke as T2/FLAIR MRI sequences showed a heart-shaped infarct, and further work-up for causes of young-onset stroke identified protein C deficiency (21 IU/dL).

During his ICU stay, the patient was treated with dual antiplatelets (Ecospirin 75 mg and clopidogrel 75 mg), statins (atorvastatin 40 mg), mechanical ventilation in pressure control mode, and antibiotics. Due to difficulties in weaning from mechanical ventilation, a tracheostomy was performed without complications, and the patient was placed on continuous positive airway pressure (CPAP). However, he developed persistent fever spikes despite no signs of aspiration. Subsequent investigations revealed a urinary tract infection (UTI) caused by multidrug-resistant *Klebsiella pneumoniae* (1,00,000 CFU/mL), complicating his clinical condition. Despite aggressive antibiotic therapy and supportive measures, the patient’s condition worsened, leading to systemic inflammatory response syndrome (SIRS), ultimately resulting in his demise.

## Discussion

BMS was first described by Spiller in 1908. This rare stroke syndrome results from infarction of the medial medulla bilaterally, usually due to occlusion of the vertebral arteries or their branches [1]. BMS, also known as Dejerine syndrome or unresponsive wakefulness syndrome with eyes open, is characterized by simultaneous infarction in both medullary pyramids [1,2]. The medulla oblongata, an essential part of the brainstem, plays a pivotal role in autonomic functions such as breathing, heart rate, and blood pressure regulation [3-5]. Infarctions in this region can lead to severe and life-threatening symptoms.

This syndrome presents a complex array of symptoms, including horizontal gaze palsy, convergence-retraction nystagmus, dysphagia, dysarthria, hemiparesis, and hyperreflexia [3-6]. Understanding medullary syndrome involves a nuanced comprehension of the neurological deficits resulting from lesions affecting the dorsal medial part of the medulla oblongata, which houses vital structures responsible for regulating autonomic functions like respiratory and cardiovascular control. This condition can often be mistaken for other neurological disorders, such as multiple sclerosis or brainstem gliomas. A hallmark of this syndrome is the V-shaped or heart-shaped appearance observed in MRI brain scans [1,7-10].

The heart-shaped infarct observed in this patient’s MRI is a unique finding that likely represents the distribution of blood flow and the anatomical configuration of the medullary arteries, serving as a radiological clue in diagnosing BMS. The medulla oblongata receives its blood supply primarily from the vertebral arteries, which merge to form the basilar artery, branching into several vessels, including the anterior spinal artery. Perforating branches from the vertebral and basilar arteries also contribute to the medulla's blood supply [5]. BMS is primarily caused by occlusion of the vertebral arteries or their branches, leading to ischemia in the medulla oblongata [6-10]. Bilateral involvement is rare due to the typically unilateral nature of vertebral artery occlusions. BMS results from bilateral infarction or ischemia affecting the paramedian branches of the vertebral arteries or the anterior spinal artery, compromising blood flow to the medial medullary region [7-13]. BMS presents with neurological deficits stemming from specific anatomical structures within the medulla, including contralateral hemiparesis or hemiplegia due to corticospinal tract involvement, ipsilateral tongue weakness or deviation due to hypoglossal nucleus involvement, and contralateral loss of proprioception and vibration sense due to medial lemniscus involvement. Dysphagia and dysarthria result from the involvement of the nucleus ambiguus [5]. Typically, in medial medullary syndrome, the tongue may deviate towards the side of the lesion, while in lateral medullary syndrome, the tongue deviation is not a hallmark feature, and other symptoms like ataxia, Horner’s syndrome, and dysphagia are more prominent [6,13].

Patients may exhibit sensory deficits and autonomic dysfunction, such as labile blood pressure and heart rate abnormalities, due to the involvement of autonomic centers in the medulla [5,6]. Differential diagnoses include other brainstem infarction, multiple sclerosis, neuromyelitis optica (NMO), acute disseminated encephalomyelitis (ADEM), central pontine myelinolysis, and leukoencephalopathies. Certain tumors can mimic the symptoms of demyelinating diseases like multiple sclerosis. Primary brain tumors, such as gliomas, meningiomas, ependymomas, and CNS lymphomas, can cause similar neurological issues like weakness, vision changes, or cognitive problems. Metastatic tumors from cancers like lung, breast, melanoma, or kidney can also spread to the brain, presenting with similar symptoms. Other conditions, like pituitary tumors or schwannomas, which affect cranial nerves, can mimic demyelination as well. These overlapping symptoms can make diagnosis challenging, and imaging like MRI is usually needed to differentiate them. Acute onset and specific MRI findings strongly support the diagnosis of BMS. The prognosis of BMS varies based on the severity of neurological deficit and associated complications like aspiration pneumonia or respiratory failure. Some patients may experience partial recovery with rehabilitation, while others may face long-term disability or mortality.

Management focuses on acute management, preventing complications, implementing rehabilitation therapies, and devising long-term strategies. Acute management includes stabilization of airway and breathing, often requiring nasogastric feeding and ventilatory support, and antithrombotic therapy with aspirin and statins to prevent thromboembolic events [6-10]. Control of risk factors such as hypertension and diabetes is crucial. Long-term management involves physical, occupational, and speech therapy to address motor and speech deficits, with regular follow-up to monitor potential complications. Early diagnosis and intervention are critical for improving outcomes. Advances in MRI technology, particularly DWI and ADC mapping, have improved the ability to diagnose brainstem infarctions accurately. This case adds to the limited pool of documented instances [1,6-12], highlighting challenges in diagnosis and management. The clinical presentation, marked by motor and cranial nerve deficits, underscores the complex neuroanatomy of the medulla. The prognosis of BMS varies, with recovery depending on the extent of infarction and the timeliness of intervention. Early recognition and treatment are pivotal in improving outcomes.

## Conclusions

BMS remains a rare but devastating neurological disorder with significant diagnostic and therapeutic challenges. The diverse range of symptoms and the condition’s ability to mimic other neurological diseases make early diagnosis difficult. However, advanced imaging techniques, particularly MRI, can provide key diagnostic clues, such as the characteristic heart-shaped infarct. While supportive care and risk factor management are essential, there remains a lack of targeted therapies to reverse the damage caused by ischemia in the medulla. Future research is needed to explore novel therapeutic strategies aimed at improving outcomes for patients affected by this rare and debilitating condition. Early recognition, prompt intervention, and a multidisciplinary approach remain crucial in optimizing patient care and potentially improving long-term outcomes.
